# Feasibility and effectiveness of endoscopic irreversible electroporation for the upper gastrointestinal tract: an experimental animal study

**DOI:** 10.1038/s41598-021-94583-w

**Published:** 2021-07-28

**Authors:** Han Jo Jeon, Hyuk Soon Choi, Bora Keum, Eun Joo Bang, Kang Won Lee, Sang Hyun Kim, Sun Young Yim, Jae Min Lee, Eun Sun Kim, Yeon Seok Seo, Yoon Tae Jeen, Hong Sik Lee, Hoon Jai Chun, Hong Bae Kim, Jong Hyuk Kim

**Affiliations:** 1 Division of Gastroenterology and Hepatology, Department of Internal Medicine, Korea University College of Medicine, 73, Goryeodae-ro, Seongbuk-gu, Seoul, 02841 Republic of Korea; 2 Department of Biosystems & Biomaterials Science and Engineering, Seoul National University, Seoul, Republic of Korea; 3 Department of Veterinary Clinical Sciences, College of Veterinary Medicine, University of Minnesota, St. Paul, MN USA

**Keywords:** Gastroenterology, Gastrointestinal system, Small intestine, Stomach

## Abstract

Irreversible electroporation (IRE) is a local non-thermal ablative technique currently used to treat solid tumors. Here, we investigated the clinical potency and safety of IRE with an endoscope in the upper gastrointestinal tract. Pigs were electroporated with recently designed endoscopic IRE catheters in the esophagus, stomach, and duodenum. Two successive strategies were introduced to optimize the electrical energy for the digestive tract. First, each organ was electroporated and the energy upscaled to confirm the upper limit energy inducing improper tissue results, including bleeding and perforation. Excluding the unacceptable energy from the first step, consecutive electroporations were performed with stepwise reductions in energy to identify the energy that damaged each layer. Inceptive research into inappropriate electrical intensity contributed to extensive hemorrhage and bowel perforation for each tissue above a certain energy threshold. However, experiments performed below the precluded energy accompanying hematoxylin and eosin staining and terminal deoxynucleotidyl transferase dUTP nick-end labeling assays showed that damaged mucosal area and depth significantly decreased with decreased energy. Relevant histopathology showed infiltration of inflammatory cells with pyknotic nuclei at the electroporated lesion. This investigation demonstrated the possibility of endoscopic IRE in mucosal dysplasia or early malignant tumors of the hollow viscus.

## Introduction

Gastrointestinal (GI) cancer is a major health concern worldwide with high incidence and mortality rates. Stomach and colorectal cancer are the 5th (5.7%) and 3rd (10.2%) most common cancers globally, representing the 3rd (8.2%) and 2nd (9.2%) leading causes of cancer-related death, respectively^1^. The cost of medical care for these cancers, especially asymptomatic cancers, is expected to increase substantially, becoming a growing social burden^2^. At the time of diagnosis, 30–50% of gastric cancers are at advanced stages, while approximately 6.3–9% show distant metastases^3^. Locally advanced unresectable and metastatic gastric cancer has a poor prognosis, with a 20–30% 5-year survival rate^4^. Thus, early detection via endoscopy and surgical resection are crucial for the management of GI cancer.

With the efficacy of perioperative chemotherapy depending on the surgical procedure remaining insufficient, the standard treatment for locally advanced gastric cancer remains unclear^5^. Advances in the development of endoscopic techniques have enabled local stent treatment, and image-guided percutaneous thermal ablation has been implemented for treating focal and locally advanced tumors since the 1990s^6,7^.

Recently, a new ablation technique called irreversible electroporation (IRE) was developed and includes electropermeabilization, which induces cell membrane perforation by an external electric field^8^. Electrical stimulation of the cell membrane changes the transmembrane potential and increases conductivity and permeability, resulting in permanent nano-sized aqueous pores on the cell membrane that eventually cause cell death^9^.

IRE possesses unique features that overcome the limitations of thermal ablation therapy, including the prevention of heat-generated damage to the surrounding normal tissue and incomplete ablation due to adjacent blood flow^10^. One distinct advantage is that the ablative effect is not affected by the heat sink effect because it uses non-thermal energy. Since tissue IRE first showed a potential ablative effect in the liver^11^, subsequent analogous studies have been reported in solid organs, including the pancreas^12^, prostate^13^, and kidney^14^.

IRE studies have mainly focused on solid organs using surgical or percutaneous methods. However, this investigation suggests the use of non-invasive IRE therapy on hollow viscus tissues. This is the first endoscopic in vivo animal IRE study performed in the upper GI tract. We aimed to assess the feasibility and effectiveness of a newly designed endoscopic IRE for the GI tract in a swine model.

## Results

### Phase 1: Escalating voltage to investigate upper threshold energy of IRE

**Figure 1:**
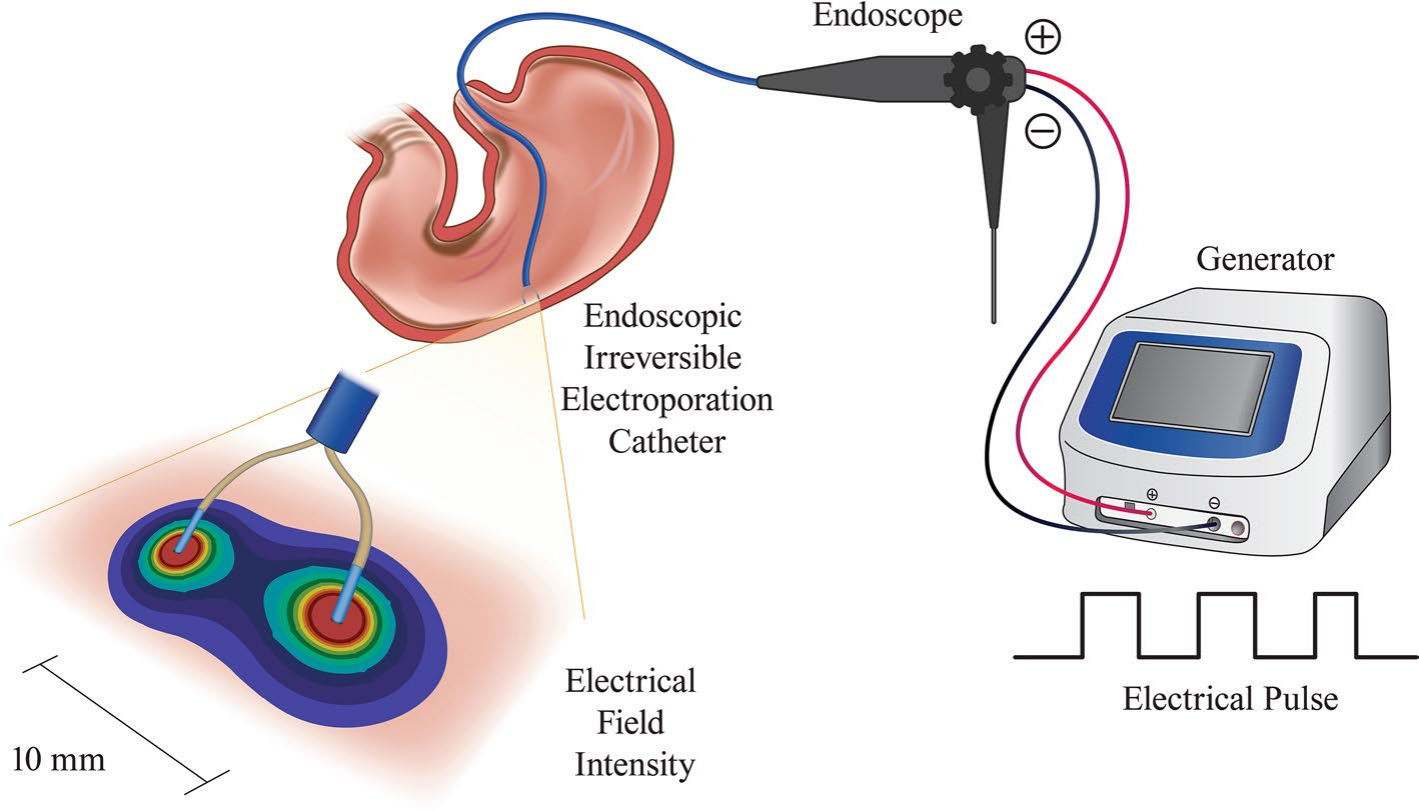
A conceptual depiction of the entire endoscopic irreversible electroporation setup for the stomach. The endoscopic ablative catheter connected to the generator is inserted into the esophagogastroduodenoscope through the channel to electroporate the target lesion.

**Figure 2:**
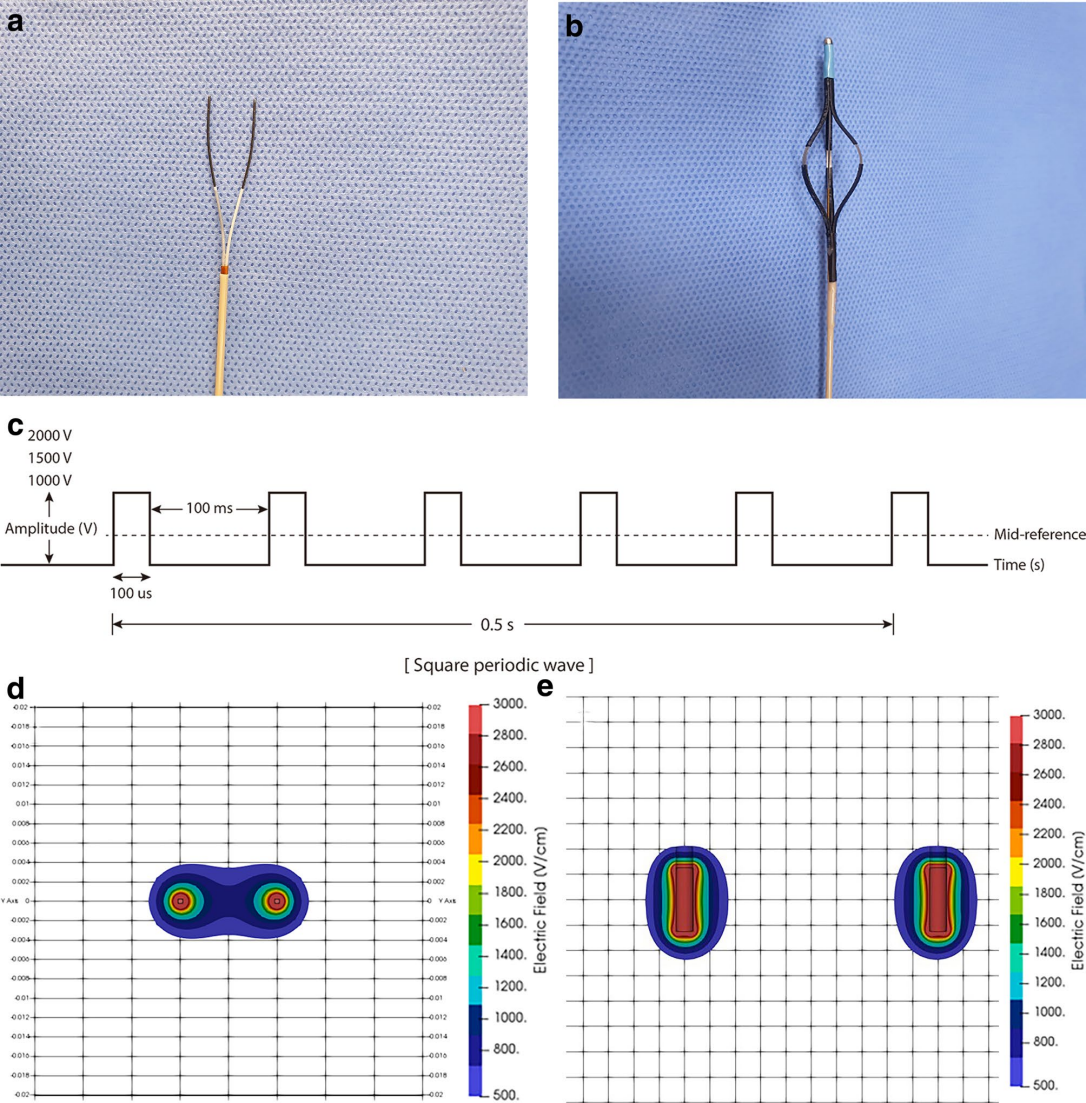
Irreversible electroporation (IRE)–related equipment specifications. Presentation of endoscopic catheters for (**a**) stomach (electrode diameter: 1.57 mm; exposed electrode length: 20 mm; distance between two electrodes: 10 mm), (**b**) esophagus and duodenum (electrode length: 5 mm; electrode width: 1.34 mm; distance between two electrodes: 20.9 mm) (1500 V/cm) with (**c**) square-shaped electrical monopolar pulse wave (pulse frequency, 10 Hz; amplitude: 1000 V, 1500 V, or 2000 V; pulse duration, 100 µs; pulse interval, 100 ms, and a fixed pulse number of 40) showing the electric field intensity of the (**d**) needle-type catheter and (**e**) basket-type catheter created by using Epocode™ (The Standard Co. Ltd.).

**Figure 3:**
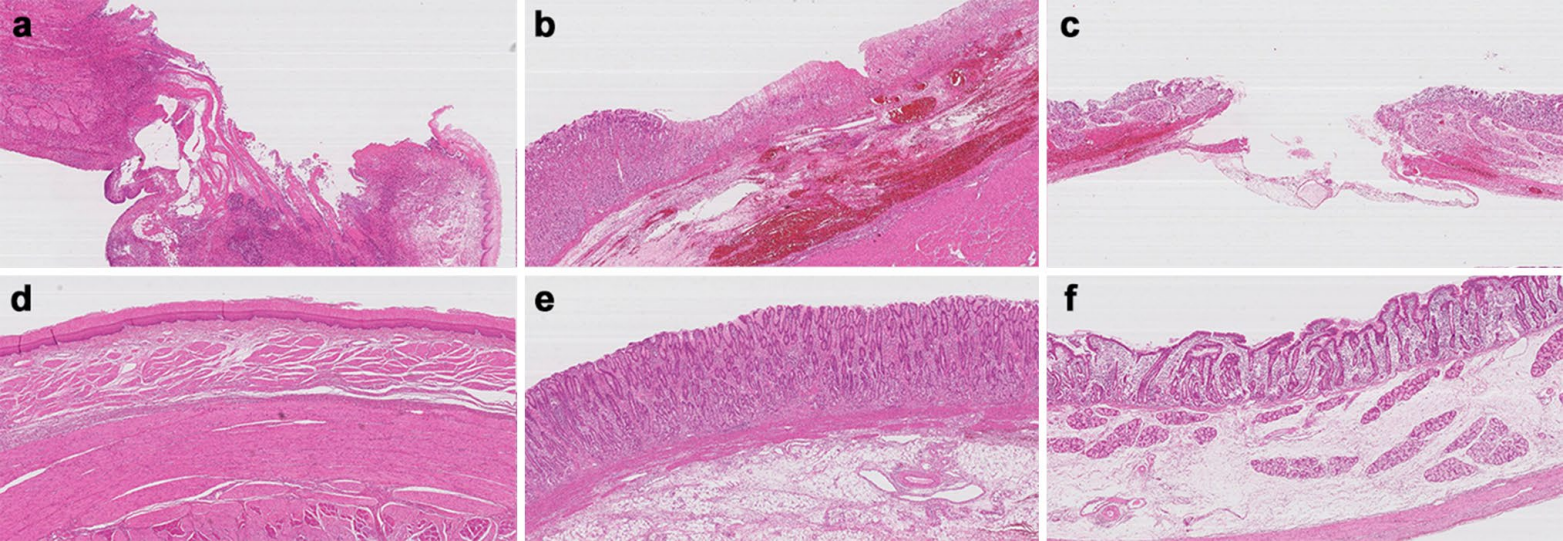
Histologic findings of adverse events in the phase 1 study. (**a**) Partial loss of the muscularis propria and surrounding inflammation with necrotic changes were exhibited at the ablation site of the esophagus treated with 2500 V (1190.5 V/cm). Hematoxylin and eosin (H&E), 20 × magnification. (**b**) Diffuse mucosal necrosis with extensive hemorrhaging in the submucosal layer were observed after ablation of the stomach at 2000 V (2000 V/cm). H&E, 40 × magnification. (**c**) The muscularis propria and submucosal layers showed complete destruction and submucosal hemorrhaging with congestion with ablation of the duodenum at 2000 V (952.4 V/cm). H&E, 20 × magnification. (**d**) normal esophagus. (**e**) normal stomach. (**f**) normal duodenum. H&E, 40 × magnification.

Figure 1 presents a drawing of the relevant endoscopic IRE procedures. A total of seven ablations were performed on each pig using the IRE equipment (Fig. 2). The duodenum and stomach were ablated at 1500 V and 2000 V, and the esophagus was ablated at 1500 V, 2000 V, and 2500 V. Based on the histopathological findings, the lowest electrical voltage to induce unfavorable events was 2500 V (1191 V/cm) in the esophagus, 2000 V (2000 V/cm) in the stomach, and 2000 V (952.4 V/cm) in the duodenum. The corresponding average electrical currents were 7.3, 4.0, and 5.8 A, respectively. Each electrical intensity above provoked hemorrhage in the stomach and perforation in the esophagus and duodenum (Fig. 3).

### Phase 2: De-escalating voltage to investigate the intensity at which the superficial layer was damaged

Depending on phase 1 findings, electrical energy values (2500 V) causing transmural damage were excluded. Accordingly, the esophagus was electroporated at 2000 V and 1500 V, while the stomach and duodenum were ablated at 1500 V and 1000 V, respectively.

The ablation depth and damaged mucosal areas were recorded according to the electrical energy applied (Table 1). The electrical voltages corresponding to muscularis mucosa damage were 1500 V (714.3 V/cm), 1000 V (1000 V/cm), and 1000 V (476.2 V/cm), while those related to submucosal damage were 2000 V (952.4 V/cm), 1500 V (1500 V/cm), and 1500 V (714.3 V/cm) in the esophagus, stomach, and duodenum, respectively.

**Table 1:** Summary of phase 2 experimental outcomes.

Pig	Location	Ablation size (mm)	Area (mm^2^)	Voltage (V)	Impedance (Z)	Current density (A/mm^2^)	Electrical field intensity (V/cm)	Electrical energy (J)		Depth
1	Esophagus	2.3 mm × 6.0 mm	13.8	2000	3194	0.0935	952.4	2.27	SM1	
	Stomach	d = 3.5 mm	9.6	1500	1593	0.0981	1500	5.65	SM2	
	Duodenum	3.7 mm × 9.0 mm	33.3	1500	865	0.259	714.3	4.72	SM1	
2	Esophagus	2.8 mm × 7.0 mm	19.6	2000	4485	0.0666	952.4	1.62	SM2	
	Stomach	d = 3.6 mm	10.2	1500	1483	0.102	1500	6.26	SM2	
	Duodenum	3.8 mm × 9.0 mm	34.2	1500	755	0.297	714.3	5.41	SM1	
3	Esophagus	2.9 mm × 6.3 mm	18.3	2000	4198	0.0711	952.4	1.73	SM2	
	Stomach	d = 3.5 mm	9.6	1500	1387	0.113	1500	6.49	SM2	
	Duodenum	3.8 mm × 8.9 mm	33.8	1500	837	0.267	714.3	4.88	SM1	
4	Esophagus	1.6 mm × 5.2 mm	8.3	1500	3036	0.0737	714.3	1.34	MM	
	Stomach	d = 2.3 mm	4.2	1000	1220	0.195	1000	3.27	MM	
	Duodenum	2.1 mm × 5.0 mm	10.5	1000	718	0.208	476.2	2.53	SM1	
5	Esophagus	1.6 mm × 5.1 mm	8.2	1500	4165	0.0538	714.3	0.98	MM	
	Stomach	d = 1.9 mm	2.8	1000	1512	0.236	1000	2.65	LP	
	Duodenum	2.2 mm × 5.8 mm	12.8	1000	742	0.201	476.2	2.44	MM	
6	Esophagus	1.5 mm × 4.9 mm	7.4	1500	3516	0.0637	714.3	1.16	LP	
	Stomach	d = 2.0 mm	3.1	1000	1449	0.223	1000	2.76	LP	
	Duodenum	1.9 mm × 5.2 mm	9.9	1000	722	0.207	476.2	2.51	MM	

*SM* submucosa, *LP* lamina propria, *MM* muscularis mucosa, *MP* muscularis propria, *d* diameter of needle-type electrode, *A* ampere, *V* volt, *J* Joule.

**Figure 4:**
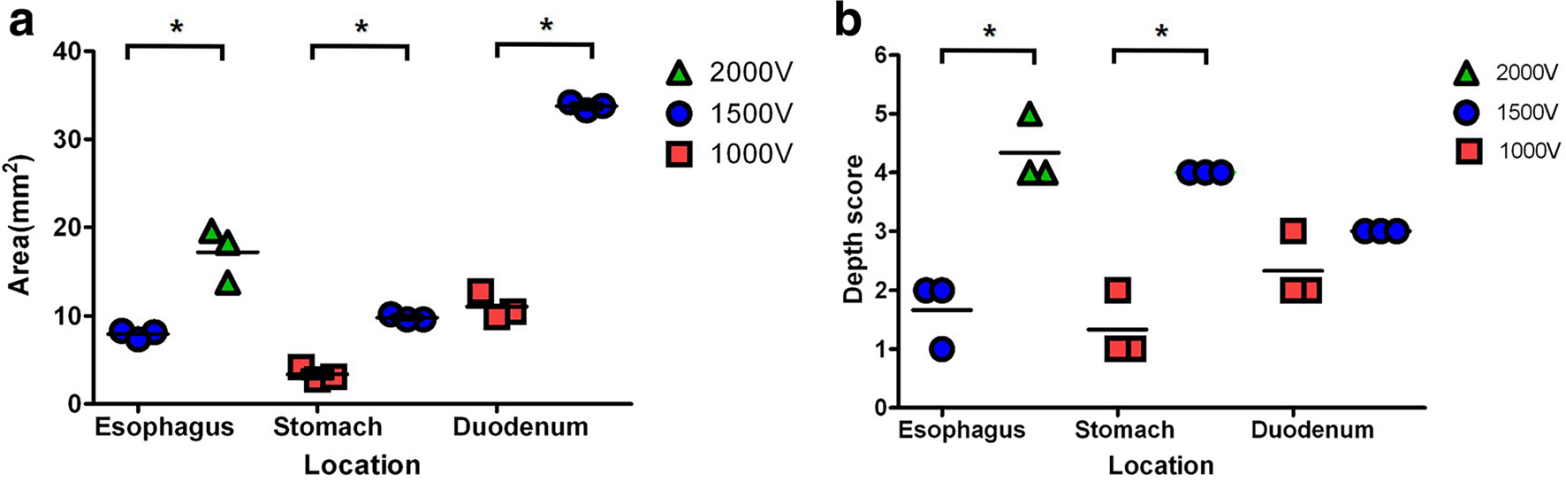
Comparison of scatter plot of electroporated area and depth by organ with electrical intensity in the phase 2 study. (**a**) Damaged surface area of the mucosa at 24 h after irreversible electroporation using the Image J program (version 1.8, National Institutes of Health). (**b**) Histologic depth scoring as follows: 1, lamina propria; 2, muscularis mucosa; 3, submucosa 1 (half above); 4, submucosa 2 (half below); 5, muscularis propria. Bar indicates median (n = 3). The Mann–Whitney U test (*p < 0.05) was used.

As Fig. 4 shows, the mucosal ablated area noted on gross tissue inspection after the application of 1500 V in the esophagus decreased significantly from a mean 18.3 mm^2^ to 8.2 mm^2^ (p = 0.0495) compared to that at 2000 V. In the stomach and duodenum, the electroporated area induced by 1000 V decreased significantly from 9.6 mm^2^ to 3.1 mm^2^ and 33.6 mm^2^ to 10.5 mm^2^, respectively (stomach: p = 0.0463; duodenum: p = 0.0495), compared to that induced by 1500 V (Supplementary Table S1).

Electroporated depth decreased from the submucosa to the muscularis mucosa at 1500 V in the esophagus and 1000 V in the duodenum. The produced lamina propria (LP) layer depth after the application of 1000 V in the stomach was shallower than that after the application of 1500 V in the submucosal layer.

### Immediate endoscopic view and tissue evaluation after 24 h

**Figure 5:**
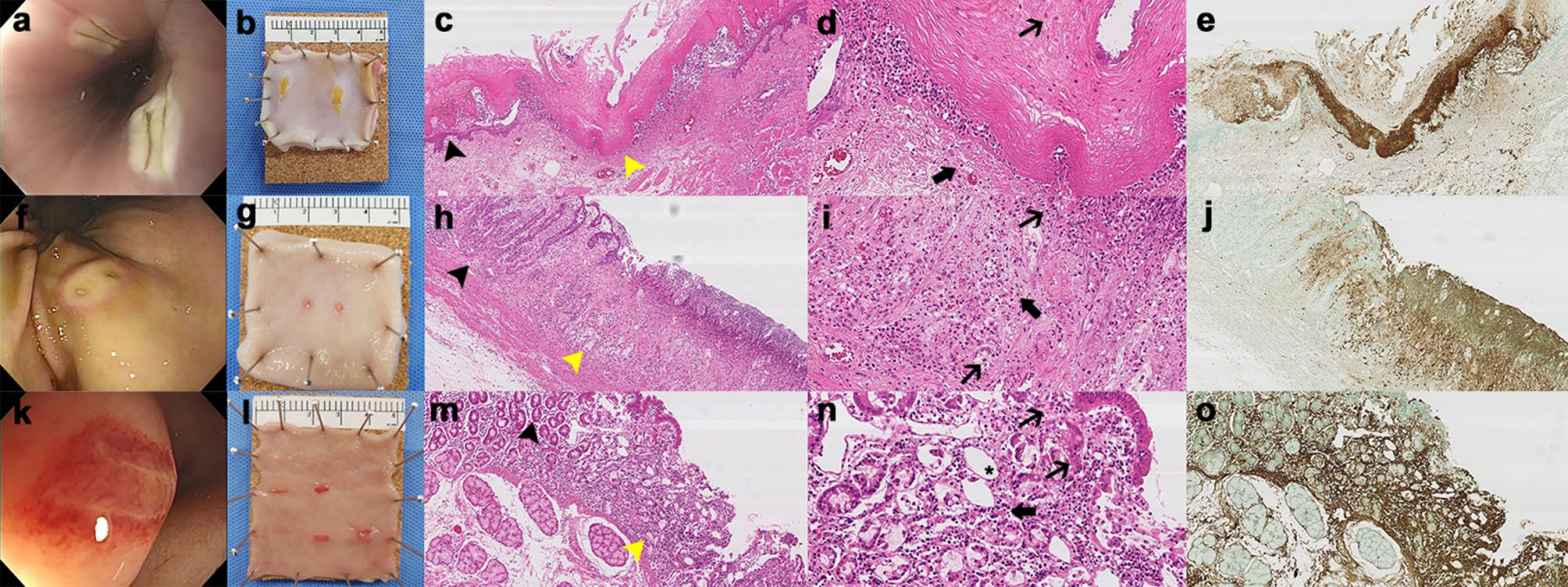
Endoscopic, gross inspection, and histopathology views after irreversible electroporation (IRE) in the phase 2 study. The first row represents the esophageal ablation effect at 1500 V (717.7 V/cm), the second row is the result of ablation of the stomach at 1000 V (1000 V/cm), and the third row shows the duodenal ablation results at 1000 V (478.5 V/cm). First column: endoscopic view immediately after IRE ablation of (**a**) esophagus, (**f**) stomach, and (**k**) duodenum. Second column: tissue gross inspection 24 h after IRE ablation of (**b**) esophagus, (**g**) stomach, and (**l**) duodenum. Third column: hematoxylin and eosin staining of electroporated (**c**) esophagus, (**h**) stomach, and (**m**) duodenum (black arrowhead; non-ablated area, yellow arrow head; ablated area). Hematoxylin and eosin (H&E), 100 × magnification. Fourth column: magnified (× 300) H&E staining of (**d**) esophagus showing epithelial karyolysis (thin arrow) and some pyknotic nuclear changes in the submucosa (thick arrow), (**i**) stomach showing a fragmented nucleus, karyorrhexis (thin arrow) and pyknotic nucleus (thick arrow), and (**n**) duodenum presenting karyolysis (thin arrow) in the form of nucleus and some pyknotic nucleus (thick arrow) with glandular atrophy (*). Fifth column: terminal deoxynucleotidyl transferase dUTP nick-end labeling assay of (**e**) esophagus, (**j**) stomach, and (**o**) duodenum. H&E: 100 × magnification.

Immediately after IRE at 1500 V in the esophagus, the mucosa in contact with the electrodes turned whitish (Fig. 5a). Unlike in the esophagus, the stomach’s electroporated mucosa showed pale snowman-shaped edema, with an erythematous peripheral rim and erosion after IRE at 1000 V (Fig. 5f). In the duodenum, the ablated mucosa exhibited a rectangular-shaped erythematous change after electroporation at 1000 V (Fig. 5k).

After 24 h, all whitish changes in the esophageal ablation area disappeared, and the electroporated mucosa showed a pattern of epithelial peeling (Fig. 5b). In contrast, the erythematous rim and edematous mucosal changes in the stomach disappeared. Only slight erosion and erythema remained (Fig. 5g). Similar to in the stomach, there was no significant difference between the ablated area and normal mucosa, except for the rectangular-shaped erythema (Fig. 5l).

### Histopathology and terminal deoxynucleotidyl transferase dUTP nick-end labeling assay

Interestingly, the application of 1500 V in the esophagus induced partial separation of the squamous epithelium and karyolysis in the ablated epithelium. Inflammatory cells infiltrated the separated mucosa (Fig. 5c), while pyknosis was observed in the LP (Fig. 5d**)**. The basal layer and basement membrane of the electroporated lesion were destroyed. On the other hand, the mucosal layer of the stomach ablated at 1000 V exhibited the loss of glandular epithelial cells with no histological damage at the submucosal layer, clear demarcation between the ablation margins (Fig. 5h) and shrunken, fragmented nucleus (Fig. 5i**)**. The duodenum ablated at 1000 V showed glandular atrophy and inflammatory cells in the LP with a preserved SM layer (Fig. 5m,n).

The esophageal stratified epithelium was terminal deoxynucleotidyl transferase dUTP nick-end labeling (TUNEL)–positive. The pyknotic nuclei in the LP and MM as well as the epithelial cytoplasm showing karyolysis were strongly stained (Fig. 5e). Likewise, the nucleus of the ablated mucosa in the stomach and duodenum was TUNEL-positive with the presence of pyknosis and karyorrhexis (Fig. 5j,o).

## Discussion

In our two consecutive experiments, we successfully demonstrated that in vivo IRE ablation of the upper GI tract was both feasible and effective. While phase 1 of our study demonstrated adverse effects such as perforation and bleeding after the application of high electrical intensity, phase 2 showed that the electroporation could be accomplished safely if optimized energy was administered.

Our study results have several important clinical implications. Endoscopic mucosal resection and endoscopic submucosal dissection (ESD) are widely accepted for treating gastric epithelial dysplasia. Nevertheless, there has been considerable debate regarding the best treatment and consistent application of ESD with risk of bleeding and perforation to the low grade dysplasia, which has a risk of cancer transformation less than 10%^15,16^. Thus, if some small neoplastic lesions are appropriately selected, we postulate that the therapeutic effect of IRE can be expected and maximized. However, IRE cannot completely replace the ESD technique in the stomach. Because the risk of metastasis and recurrence is high in cases of early gastric cancer that are larger than 2 cm or accompanied by ulceration, preferentially applying IRE is considered difficult^17^.

Before argon plasma coagulation (APC) was applied to the digestive tract as an adjunctive ablation therapy for GI endoscopy, several experimental investigations were conducted on the effect of APC on depth according to gas flow or power range for safety^18,19^. Since then, APC has clinically confirmed the safety and effectiveness of EGC treatment in cases in which it is difficult to apply endoscopic mucosal resection^20^. A recent study reported that APC can be applied as its complication rate is lower than that of ESD in low-grade dysplasia lesions less than 2 cm and showed cost-effectiveness^21^. Therefore, it would be beneficial if an appropriate lesion could be selected with the adequate IRE parameters that are safe for the GI tract.

Recent advances in the IRE technique have provided an opportunity to improve the treatment and management of advanced malignancy. Therefore, IRE can also be attempted as a palliative treatment for obstructive lesions caused by locally advanced tumors that cannot be surgically resected^22,23^. Additionally, the unique IRE characteristics compared to those of coagulation therapy such as APC and RFA preserve organ structures such as nerves and vessels in the non-thermal area, which can reduce patient complications and maintain function, thereby improving quality of life^24,25^. Therefore, we believe that this new ablation technique paves the way for a new minimally invasive endoscopic treatment for GI malignancies, suggesting its potential clinical application for the treatment of GI neoplasms.

There are currently two standard ablative methods for the GI tract: RFA and APC. In RFA, heat is dissipated from the tissues around the vessels (diameter > 3 mm) due to blood flow, thus inducing the heat sink effect and increasing recurrence rates^26^. In contrast, short electrical pulses during IRE circumvent the heat sink effect while selectively preserving vital structures such as vessels, nerves, and ducts^27^. Thus, IRE is gaining attention as a therapeutic alternative to pre-existing ablation. To date, limited preliminary IRE studies have focused on the hollow viscus of the GI tract: one of the esophagus^28^, two of the stomach^29,30^, two of the small bowel^31,32^, one of the colon^33^, and one of the rectum^34^.

We observed that the electroporated depth differed among tissue types, even under the same electrical intensity, as reported in previous RFA studies^35^. Our experimental results showed that the esophagus required a higher energy intensity than the stomach or duodenum for the identical layer to be damaged. According to impedance data, the relatively small damage induced in the esophagus is interpreted as its average resistance being lower than that of the stomach and duodenum, decreasing the electrical conductivity and current flow.

Another finding was that the damage depth was shallower in the duodenum than in the stomach after the application of the same voltage, which is probably attributable to the wider electrode area and longer electrode interval distance inducing energy dispersion. The stronger the intensity of the electrical energy applied, the deeper the tissue damage in the same organ. According to Ohm’s law, the greater the amount of current flowing through the tissue, the stronger the electrical energy in tissues with the same impedance^36^.

Theoretically, IRE uses non-thermal energy, but secondary Joule heating also occurs above a certain energy threshold. We confirmed here that hemorrhaging occurred in the SM layer during ablation at 2000 V/cm in the stomach. According to a previous IRE study on vessels, a voltage of 115 V (3800 V/cm), pulse length of 100 µs, frequency of 10 Hz, and total of 10 pulses were set as the maximal conditions that did not induce thermal energy production, at a 3-mm carotid artery in the rat^37^. Therefore, it was assumed that thermal damage occurred beyond the referred maximal conditions because the vessel of the porcine submucosa is smaller than 1 mm^38^.

According to recent IRE heat generation studies, although the initial pulses of IRE are non-thermal, the temperature increases as the number of delivered pulses increases^39,40^. As a result, tissue resistance decreases and electrical current increases as the temperature increases during the IRE procedure. The preceding result is consistent with that of a study that reported increased electrical conductivity during IRE^40,41^. Studies have demonstrated that the increase in conductivity was caused by increased heat and cellular permeability as the delivered energy increased.

The present catheter-induced IRE investigation showed perforations in the esophagus and duodenum. In contrast, previous IRE studies performed on the biliary tract and ureter did not show perforations^42,43^. Our opposite results might have been attributable to the small electrode area (6.7 mm^2^) and electrical over-flowing current in the tissues between electrodes and thus thermal injury due to Joule heating. The current density of 1.13 A/mm^2^ (Joule heat) for the applied voltage of 2500 V in our study was approximately 3.3 times that of the other study^42^.) Converted to Joule heating, the electrical energy we applied to the esophagus (3.5 J) and duodenum (9.8 J) might have been sufficient to perforate the tissues. This originated from a geometrical electrode difference, while the thermal damage resulted from excessive current density.

Therapeutic endoscopists will be interested in the destruction of neoplastic lesions in the mucosal layer. Since the submucosa is responsible for the regeneration of vessels, nerves, and tissues, previous RFA studies set the criteria for the appropriate ablative energy based on histology confirming the absence of damage to the submucosa^44^. Accordingly, 714.3–952.4 V/cm in the esophagus, 1000–1500 V/cm in the stomach, and 476.2–714.3 V/cm in the duodenum could be considered suitable electrical intensities to target intramucosal lesions with minimal damage to the submucosa.

This study has several limitations, one of which is that the ablation was performed in normal tissues. Tumor cells and tissues have different structures and are known to be more vulnerable to heat and electrical environments^45^. Additionally, the junctions of tumor cells are leaky and have a lower impedance than those of normal cells^46,47^. Therefore, the damaged tissue depth, damaged area, and required electrical energy for ablation of a specific layer may differ in tumor environments, and it can be predicted that tumor tissue ablation would be attempted at a lower electrical field than normal tissue ablation. Second, the number of animals per group was reduced to three because the primary goal was to examine IRE feasibility and safety under various conditions. Although the number of experiments was small to reveal statistical significance regarding measured depth and area, there was no difficulty in confirming the tendency because of the accuracy and precision revealed in the scatter plot. Third, we assumed that the energy supplied from the generator would be entirely transformed into tissue damage in this study. However, there may be a difference between the energy supplied from the generator and the energy actually applied to the tissue. This is because energy loss occurs through heat generation due to tissue impedance during electroporation or due to improper contact between the tissue and electrodes. Therefore, to reduce energy loss, it may be more accurate to measure the actual current flowing in the tissue and compare it with the damage.

In conclusion, endoscopic IRE in the esophagus, stomach, and duodenum was feasible, effective, and safe. Our research demonstrated that endoscopic IRE could be a new ablation therapy and option for upper GI tract neoplasms. However, further histological assessments of IRE, including ablated width, depth, and adverse events, should be preceded by appropriate electrical parameters before its clinical application.

## Methods

### Ethics statement

All animal experiments were approved by the Institutional Animal Care and Use Committee of Korea University College of Medicine (nos. KOREA-2019-0146, KOREA-2021-0017) and conducted in accordance with the relevant animal guidelines. All experimental procedures and data acquisition, interpretation, and analysis regarding live animals were also performed following the Animal Research: Reporting of In Vivo Experiments (ARRIVE) guidelines.

### Study design

The study consisted of two consecutive phases. Phase 1 comprised an experiment to determine the detrimental IRE energy levels for the GI tract. The esophagus and duodenum were sequentially ablated at 3-cm intervals from the esophagogastric junction and pylorus, respectively, and the stomach was electroporated in the antrum. The selection of 1500 V as the initial baseline and upscaling at 500-V intervals were chosen based on previous studies^42,48^.

The appropriate energy of IRE to ablate the superficial layer were investigated in phase 2. The selected voltage was 500 V less than the undesirable voltage selected in phase 1, and the electrical voltage was gradually lowered in 500-V decrements until the intensity that electroporates the mucosal layer was identified.

### Experimental animal model of IRE

To prepare the endoscopic IRE animal model, eight female YLD pigs (12 wk, 40 ± 2 kg; XP-bio Inc, Gyeonggi-do, Korea) were acclimated to the animal facility (one pig/cage, 50% relative humidity, 23 °C temperature, 12-h light/dark cycle) for 7 days and fed lab hog chow no. 38075 (Cargill Agri Purina Inc, Gyeonggi-do, Korea).

### Endoscopic IRE procedure

On the day of the experiment, the pigs were subjected to general anesthesia (induction: azaperone 2–8 mg/kg, xylazine 1–3 mg/kg, alfaxalone 2–6 mg/kg, atropine 0.5 mg/kg; maintenance: 2% isoflurane) with additional drugs (enrofloxacin 5 mg/kg, ketoprofen 3 mg/kg) and an endotracheal tube (6.5 Fr, Henan Tuoren Medical Device Co., Henan, China) was inserted. Following intubation, a 25-cm-long overtube (Guardus®, US Endoscopy, Mentor, Ohio, USA) was inserted into the esophagus, and endoscopy (GIF-Q260; Olympus, Tokyo, Japan) was performed through the overtube. The duodenum, stomach, and esophagus were sequentially ablated using the IRE catheter (Fig. 1).

### IRE-associated equipment: IRE catheters and pulse generator

We prepared two types of catheters designed for IRE: the needle-type catheter (EPO-E1; The Standard Co. Ltd., Gyeonggi-do, Korea), which was made with NiTi alloy wire for the stomach (Fig. 2a), and the basket-type catheter (EPO-G2; The Standard Co. Ltd.) for the esophagus and duodenum, which had three wheels, two of which consisted of rectangular electrodes (Fig. 2b). They all were capable of approaching the area to be ablated through a channel of the endoscopy. The pulse generator used in the IRE procedure was a BTX Gemini X2 (BTX^®^, Holliston, USA), which produced an electrical monopolar pulse with a maximum voltage of 3 kV (Fig. 2c). The impedance between the two electrodes was measured before applying electrical energy to the targeted area. To evaluate the current over the tissues, a current probe (TCPA300; Tektronix, Beaverton, OR, USA) connected to an oscilloscope (TDA3044B; Tektronix, Beaverton, OR, USA) was clipped to an electrical wire from the pulse generator.

### Simulated electrical field intensity of IRE

The electrical field distribution was acquired by calculating Laplace’s equation:

1 $${\nabla }^{2}\phi =0$$

where $\nabla$ is the gradient operator and $\phi$ is the electric potential (volt). The boundary conditions were $\phi \left(0\right)=0 \,{\rm and}\, \phi ({\phi }_{0})={V}_{0}$. The space between the tissue and air and between the catheter and air was considered insulated. The electrical field produced by the needle-type (Fig. 2d) and basket-type catheters (Fig. 2e) was simulated before the experiment using Epocode™ (The Standard Co. Ltd.), which was developed by OpenFOAM (Opensource Field Operation And Manipulation), an open-source computational fluid analysis-oriented software.

### Histologic analysis and immunochemistry

Endoscopy confirmed the absence of bleeding or perforation in the lesions at 24 h post-ablation and then pigs were sacrificed. After the electroporated tissues were fixed in 10% formalin solution for 24 h. Next, the fixed tissues were mounted in paraffin and sliced into 3-µm-thick sections and analyzed using hematoxylin and eosin (H&E) staining. An additional TUNEL assay (ApopTag^®^ Peroxidase In Situ Apoptosis Detection Kit, S7100, Millipore, Gaithersberg, MD, USA) was performed to assess tissue apoptosis. After fixation, the presence of brown stained cells was considered positive TUNEL assay findings.

### Image analysis of electroporated surface area and ablation depth

The ablation area was measured using Image J software (version 1.8; National Institutes of Health, Bethesda, Maryland, USA). Adverse events were defined as extensive submucosal hemorrhage, muscularis propria damage, or perforation. Ablation depth was recorded as the deepest histological damaged layer. H&E- and TUNEL-stained tissue specimen slides were analyzed using a slide scanner (Leica SCN400; Leica Microsystems, Wetzlar, Germany) and an image viewer (Leica SCN400 Image Viewer; Leica Microsystems, Wetzlar, Germany).

### Statistical analysis

The scatter plots of damaged area and depth were represented using GraphPad PRISM^®^ (version 5.1; GraphPad Software, San Diego, California, USA). Non-parametric continuous and ordinal variables are expressed as median and interquartile range using SPSS^®^ (version 24.0; IBM Corp, Armonk, N.Y., USA). The Mann–Whitney U test was used to compare depth and area by electrical intensity. Values of p < 0.05 were considered statistically significant.

## Supplementary Information

Supplementary Information.

## Data Availability

All relevant data are presented in the manuscript.
