# Supplementary material for: Feasibility and effectiveness of endoscopic irreversible electroporation for the upper gastrointestinal tract: an experimental animal study
Source: Sci Rep. 2021 Jul 28;11:15353. doi: 10.1038/s41598-021-94583-w (PMC8319327; doi:10.1038/s41598-021-94583-w)
Supplement: Supplementary file 1 — Supplementary Information. [file 41598_2021_94583_MOESM1_ESM.docx]

**Supplementary 1.**

Table S1 Damaged surface area and tissue depth analysis using Mann-Whitney U test regarding tissue type and electric field strength.

| Damage | Esophagus (N=6) | | P | Stomach (N=6) | | P | Duodenum (N=6) | | P |
| --- | --- | --- | --- | --- | --- | --- | --- | --- | --- |
|  | 1500V (N=3) | 2000V (N=3) |  | 1000V (N=3) | 1500V (N=3) |  | 1000V (N=3) | 1500V (N=3) |  |
| Area, Median, IQR | 8.2 (7.76 - 8.24) | 18.3 (16.05 - 18.95) | 0.0495 | 3.1 (2.95 - 3.65) | 9.6 (9.6 - 9.9) | 0.0463 | 10.5 (10.19 - 11.65) | 33.6 (33.8 - 34) | 0.0495 |
| Depth, Median, IQR | 2 (1.5 - 2) | 4 (3.5 - 4) | 0.0431 | 1 (1 - 1.5) | 4 (4 - 4) | 0.0339 | 2 (2 - 2.5) | 4 (3.5 - 4) | 0.114 |

IQR: interquartile range.

Depth score 1: lamina propria; score 2: muscularis mucosa; score 3: submucosa 1; score 4: submucosa 2; score 5: muscularis propria
